# Machine Learning for Early Prediction of Major Adverse Cardiovascular Events After First Percutaneous Coronary Intervention in Patients With Acute Myocardial Infarction: Retrospective Cohort Study

**DOI:** 10.2196/48487

**Published:** 2024-01-03

**Authors:** Pin Zhang, Lei Wu, Ting-Ting Zou, ZiXuan Zou, JiaXin Tu, Ren Gong, Jie Kuang

**Affiliations:** 1 Jiangxi Provincial Key Laboratory of Preventive Medicine School of Public Health Nanchang University Nanchang China; 2 School of Public Health and Management Nanchang Medical College Nanchang China; 3 Department of Cardiology The Second Affiliated Hospital of Nanchang University Nanchang China

**Keywords:** acute myocardial infarction, percutaneous coronary intervention, machine learning, early prediction, cardiovascular event

## Abstract

**Background:**

The incidence of major adverse cardiovascular events (MACEs) remains high in patients with acute myocardial infarction (AMI) who undergo percutaneous coronary intervention (PCI), and early prediction models to guide their clinical management are lacking.

**Objective:**

This study aimed to develop machine learning–based early prediction models for MACEs in patients with newly diagnosed AMI who underwent PCI.

**Methods:**

A total of 1531 patients with AMI who underwent PCI from January 2018 to December 2019 were enrolled in this consecutive cohort. The data comprised demographic characteristics, clinical investigations, laboratory tests, and disease-related events. Four machine learning models—artificial neural network (ANN), k-nearest neighbors, support vector machine, and random forest—were developed and compared with the logistic regression model. Our primary outcome was the model performance that predicted the MACEs, which was determined by accuracy, area under the receiver operating characteristic curve, and F1-score.

**Results:**

In total, 1362 patients were successfully followed up. With a median follow-up of 25.9 months, the incidence of MACEs was 18.5% (252/1362). The area under the receiver operating characteristic curve of the ANN, random forest, k-nearest neighbors, support vector machine, and logistic regression models were 80.49%, 72.67%, 79.80%, 77.20%, and 71.77%, respectively. The top 5 predictors in the ANN model were left ventricular ejection fraction, the number of implanted stents, age, diabetes, and the number of vessels with coronary artery disease.

**Conclusions:**

The ANN model showed good MACE prediction after PCI for patients with AMI. The use of machine learning–based prediction models may improve patient management and outcomes in clinical practice.

## Introduction

Acute myocardial infarction (AMI) is a common clinical acute and severe disease with rapid onset, rapid progression, and high mortality [1-3]. In 2017, there were approximately 695,000 new cases of AMI in the United States, and it is estimated that 325,000 people will have recurrent events [4]. There are approximately 500,000 new cases of AMI in China every year, and 2.5 million patients have a history of myocardial infarction [5]. As technology has advanced, percutaneous coronary intervention (PCI) has become the primary approach for treating AMI. Although PCI can significantly reduce the fatality rate of AMI, the rate of major adverse cardiovascular events (MACEs) among patients after PCI is still very high, which seriously affects the clinical outcomes of patients [6-10]. A study by Copeland-Halperin et al [11] showed that the incidence of MACEs in patients with AMI one year after PCI was 17.8% [11].

Identifying patients with AMI undergoing PCI who are at high risk of MACEs may help clinical decision-making incorporate timely measures to improve clinical outcomes. Some studies, such as Global Registry of Acute Coronary Event [12], Thrombolysis in Myocardial Infarction Risk [13,14], and Acute Catheterization and Urgent Intervention Triage StrategY-PCI [15], as well as studies that generated the Mayo Clinic PCI Risk and the China Acute Myocardial Infarction scoring systems, have explored the risks after PCI [16]. Despite these advances, individualized prediction of MACEs remains challenging with low specificity and positive predictive accuracy, and most of the methods rely on traditional parameter models, such as logistic regression, to screen for variables and build a series of risk-scoring models.

In recent years, machine learning methods that rely on a strong self-learning capability, such as random forest (RF), k-nearest neighbors (KNN), support vector machine (SVM), and artificial neural network (ANN) have become increasingly prevalent in prognostic prediction [1,13,17,18]. By calling various functions, these models can extract and integrate information from all kinds of complex data to make better predictions. A study of a consecutive cohort of patients with hypertrophic cardiomyopathy (HCM) presented a machine learning–based model to identify individual patients with HCM at high risk of developing advanced heart failure symptoms. The results showed that the 5-year risk prediction of progressive heart failure in patients with HCM can be estimated [19].

We found that machine learning models, such as RF, ANN, SVM, and KNN, perform well in clinical prognosis prediction research. Thus, this study sought to develop a machine learning–based model, integrating clinical, anatomical, and laboratory features, to predict MACEs in patients who have recently been diagnosed with AMI after their first PCI and improve overall patient outcomes by implementing earlier management.

## Methods

### Study Design, Setting and Participant Selection

This retrospective cohort study was conducted at the Department of Cardiovascular Medicine, the Second Affiliated Hospital of Nanchang University (a teaching tertiary hospital), in Jiangxi Province, China. We collected electronic medical records of patients with AMI who underwent PCI for the first time from January 2018 to December 2019. These patients were followed up through December 2021.

The inclusion criteria of the participants were as follows:

- The patient was ≥18 years of age.
- This was the patient’s first clinically diagnosed AMI (clinical evidence of AMI as evident from the detection of a rise or fall of cardiac troponin values and at least one of the following symptoms of myocardial ischemia: symptoms of acute myocardial ischemia, new ischemic electrocardiogram (ECG) changes, and development of pathological Q waves.
- PCI was performed for the first time at this hospital.
- Among the left main artery, left circumflex branch, left anterior descending branch, and right coronary artery, at least one had stenosis ≥50%.
- Complete medical records and follow-up data were available.

The following exclusion criteria were applied:

- History of PCI and coronary artery bypass grafting treatment
- Complications from other heart diseases requiring surgical procedures, such as heart bypass
- Recent active bleeding
- An intracerebral mass or an aneurysm

We adopted the “Guidelines for Developing and Reporting Machine Learning Predictive Models in Biomedical Research” to guide the reporting of our study [20].

### Data Collection, Definition of Outcomes, and Predictor Variables

Data were collected from electronic health records, including demographic characteristics, clinical investigations, the first laboratory tests, and disease-related events. MACEs were defined as cardiomyopathies (excluding infectious, familial, alcohol, and drug-related cardiomyopathies), hypertensive heart disease, recurrent myocardial infarction, heart failure, sudden cardiac death, revascularization, malignant arrhythmia, and stent thrombosis [21]. Abnormal Q waves were identified by the clinician based on ECG results. Left ventricular ejection fraction (LVEF) was defined as normal (more than 50%), mildly abnormal (40% to 50%), moderately abnormal (30% to 40%), and severely abnormal (less than 30%) [22]. According to the number of diseased coronary vessels and implanted stents, they were classified as I, II, III, and IV.

### Ethics Approval

This study was reviewed and approved by the Second Affiliated Hospital of Nanchang University Medical Ethics Committee (No. Review 2017 No. (098)).

### Data Preprocessing for Machine Learning Model Development

All analyses were performed with R software (version 4.0.1; R Core Team). The patients were randomly assigned to training (n=953, 70%) and testing (n=409, 30%) data sets by calling the createDataPartition function using the random number method, and chi-square tests showed that there was no statistical difference between them (*χ2*_1_=2.169; *P*=.14). We developed machine learning models using the training data set. We analyzed the missing and out-of-range values with imputation methods. We used multiple imputation with chained equations to assign any missing predictor values [23]. The imputation processes were performed separately in the training and testing sets after the data were split. To improve the accuracy of the machine learning models and increase the speed of finding the optimal solution by gradient descent, we standardized and normalized all input variables before the model was built. To alleviate the problem of imbalanced classification samples, we adopted the random oversampling method. We used the ROSE package in R to generate new balanced training data. After random oversampling, the number of patients with MACE in the training data sets changed from 186 to 471.

### Predictor Selection for Model Development

The model was built using demographic information (age and sex), personal comorbidities (diabetes and peripheral arterial disease), preoperative PCI (LVEF, the number of diseased vessels, and abnormal Q waves), serological examination (beta 2 microglobulin, B-type brain natriuretic peptide, glucose, serum creatinine clearance, and estimated glomerular filtration rate), and the characteristics of PCI (the number of implanted stents; n=65; Table S1 in Multimedia Appendix 1). A total of 12 variables with significant differences in the univariate analysis were included in the model development (Table 1).

**Table 1:** Baseline characteristics of the study patients (N=1362).

Variables		MACE^a^ (n=252)	Non-MACE (n=1110)	*P* value	
**Age, n (%)**					.04
	<65	101 (40.08)	543 (48.92)		
	65	94 (37.30)	332 (29.91)		
	75	57 (22.62)	235 (21.17)		
**Diabetes, n (%)**					.04
	Yes	75 (29.76)	261 (23.51)		
	No	177 (70.24)	849 (76.49)		
**Vascular disease, n (%)**					.04
	Yes	111 (44.05)	569 (51.26)		
	No	141 (55.95)	541 (48.74)		
**Abnormal Q wave, n (%)**					.04
	Yes	125 (49.60)	480 (43.24)		
	No	127 (50.40)	630 (56.76)		
**LVEF^b^, n (%)**					.005
	>50%	167 (66.27)	832 (74.95)		
	40%-50%	57 (22.62)	188 (16.94)		
	30%-40%	19 (7.54)	65 (5.86)		
	<30%	9 (3.57)	25 (2.25)		
**Vessels with coronary artery disease, n (%)**					<.001
	Ⅰ	45 (17.86)	288 (25.95)		
	Ⅱ	75 (29.76)	370 (33.33)		
	Ⅲ	123 (48.81)	418 (37.66)		
	Ⅳ	9 (3.57)	34 (3.06)		
**Implanted stent number, n (%)**					.004
	No stent	10 (3.97)	40 (3.60)		
	Ⅰ	106 (42.06)	594 (53.51)		
	Ⅱ	84 (33.33)	301 (27.12)		
	Ⅲ	37 (14.68)	114 (10.27)		
	≥Ⅳ	15 (5.95)	61 (5.50)		
Brain natriuretic peptide (pg/μL), mean (SD)		684.36 (997.90)	518.27 (773.65)	.01	
Serum creatinine clearance (mL/min), mean (SD)		65.19 (30.18)	71.87 (44.35)	.02	
EGFR^c^ (ml/min), mean (SD)		75.68 (28.92)	80.55 (31.82)	.03	
Beta 2 microglobulin (mg/L), mean (SD)		3.23 (3.61)	2.72 (5.51)	.03	
Glucose (mmol/L), mean (SD)		7.22 (3.32)	6.68 (3.00)	.02	

^a^MACE: major adverse cardiovascular events.

^b^LVEF: left ventricular ejection fraction.

^c^EGFR: estimated glomerular filtration rate.

### Model Testing and Performance Evaluation

Based on a previous application of the model [24], the parameter range of the model was preset, and the GridSearchCV function was used to select the optimal parameters of each machine learning model.

To minimize potential overfitting in the above machine learning models, we called the trainControl function in the caret package of R language for 7-fold cross-validation during the development process. The model performance was assessed for accuracy, recall, precision, area under the receiver operating characteristic curve (AUC), and *F*_1_-score in the testing data set. We identified the important predictors through importance analysis of the variables. Logistic regression analysis was used to compare the absolute value of the coefficients of variables; RF was used to measure the importance of features by calculating information gain through entropy; and the ANN method was used to calculate the relative importance of variables based on the generalized weight method.

### Statistical Analysis

The following R packages for machine learning approaches were used: caret, randomForest, and neuralnet. Baseline characteristics were compared with the Wilcoxon rank sum test for continuous variables and the chi-square test for categorical variables. We considered *P*<.05 (2-sided) to be statistically significant.

## Results

**Figure 1:**
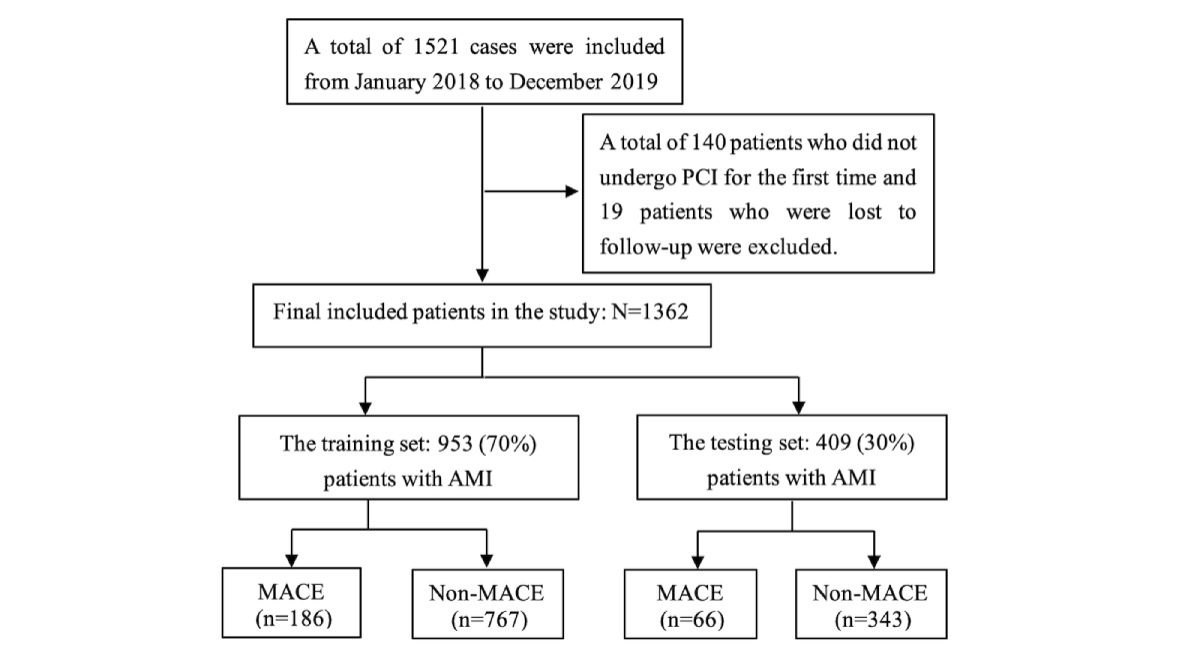
Flowchart for patient enrollment. AMI: acute myocardial infarction; MACE: major adverse cardiovascular event.

**Figure 2:**
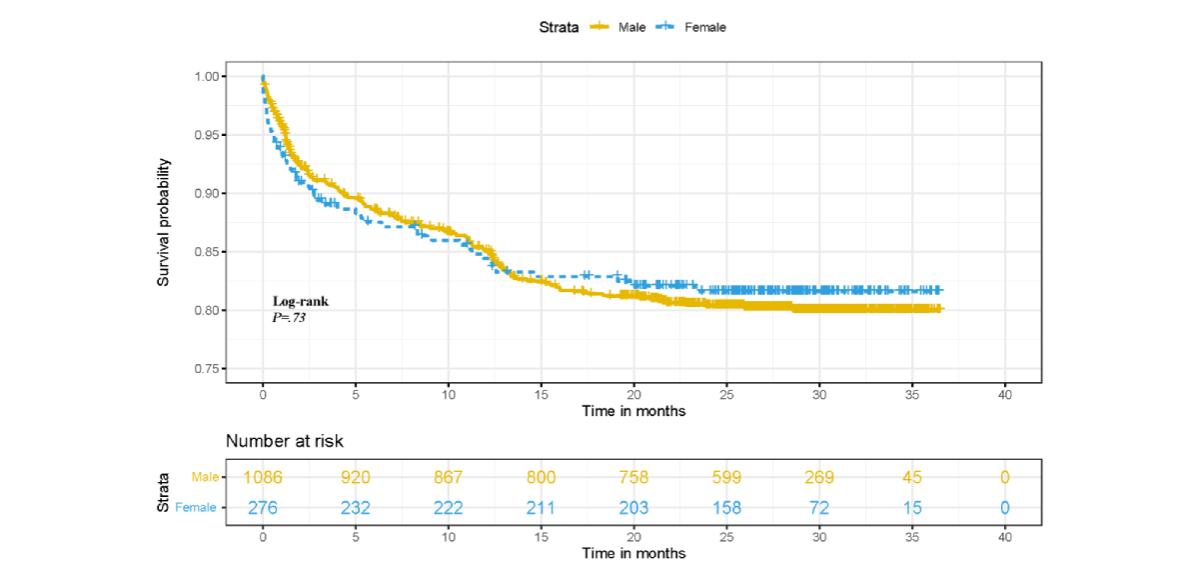
Prognostic survival curve of patients with acute myocardial infarction undergoing percutaneous coronary intervention.

A total of 1531 patients were screened; 140 patients who did not undergo PCI for the first time were excluded; 19 patients were lost to follow-up; and 1362 patients who were successfully followed up were included in this analysis (Figure 1). The mean follow-up time was 28.0 (SD 11.0) months (median 29.9 months). A total of 252 MACEs were observed, including 128 cases of recurrent myocardial ischemia and 117 cases of myocardial infarction and reinfarction. The positive rates of MACEs were 4.63%, 11.38%, 14.54%, and 18.50% at 30 days, 6 months, 1 year, and 3 years after PCI, respectively. MACEs occurred in 203 (18.7%) male patients and 49 (17.8%) female patients. As shown in Figure 2, the survival rate of the sample population decreased rapidly in the first 3 months after PCI, especially 30 days after PCI, and there was no difference in the log-rank test of the survival curve between male and female patients.

Table 1 shows the baseline characteristics of the MACE group and the non-MACE group. Age, diabetes, peripheral and cerebrovascular history, LVEF, abnormal Q wave, the number of vessels with coronary artery disease, the number of implanted stents, brain natriuretic peptide, serum creatinine, estimated glomerular filtration rate, beta 2 microglobulin, and glucose were significantly different between the 2 groups (*P*<.001). The nonsignificant differences in variables between the 2 groups are shown in Table S1-S6 in Multimedia Appendix 1.

Table 2 shows the performance of the 3 models with 7-fold cross-validation. ANN, KNN, SVM, RF, and logistic regression exhibited the best to worst performance in terms of their AUC, accuracy, recall, and *F*_1_-score. However, KNN performed best in terms of precision. The average accuracy, recall, precision, AUC, and *F*_1_-score of the ANN model were 80.52%, 81.33%, 69.94%, 83.68%, and 79.47%, respectively.

**Figure 3:**
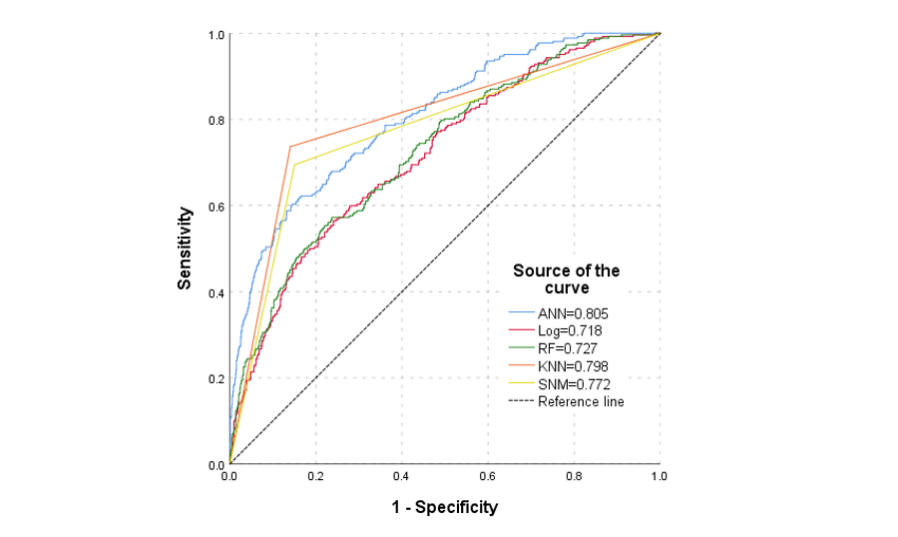
The area under the receiver operating characteristic (ROC) curve of artificial neural network (ANN), random forest (RF), k-nearest neighbors (KNN), support vector machine (SVM), and logistic regression models.

In the testing data set, the ANN model showed a higher AUC than RF and logistic regression. Figure 3 shows that the AUCs of the ANN, RF, KNN, SVM, and logistic regression models were 0.805, 0.798, 0.772, 0.727, and 0.718, respectively; the average accuracy for the above 3 models was 0.821, 0.741, and 0.729, respectively, and the average *F*_1_-scores were 0.804, 0.722, and 0.709, respectively.

The 10 most important predictors in the ANN model are shown in Table 3. These were LVEF (0.27), the number of implanted stents (0.14), age (0.13), diabetes (0.10), the number of vessels with coronary artery disease (0.09), vascular disease (0.08), brain natriuretic peptide (0.05), glucose (0.05), beta 2 microglobulin (0.04), and abnormal Q wave (0.02).

**Table 2:** Comparison of models for predicting major adverse cardiovascular events based on 7-fold cross-validation.

Models	Accuracy, mean (SD)	Recall, mean (SD)	Precision, mean (SD)	AUC^a^, mean (SD)	*F*_1_-score, mean (SD)
Logistic regression	72.37 (2.05)	67.33 (8.42)	59.62 (8.34)	73.52 (2.37)	71.11 (6.01)
K-nearest neighbors	81.44 (2.22)	80.23 (1.56)	70.22 (7.23)	81.87 (3.32)	77.95 (5.70)
Support vector machine	74.91(3.03)	80.03(1.76)	65.94 (7.02)	78.68 (1.82)	76.41 (5.92)
Random forest	73.44 (1.58)	71.23 (1.56)	61.22 (7.23)	74.87 (2.12)	71.92 (6.30)
Artificial neural network	80.52 (1.13)	81.33 (0.56)	69.94 (7.02)	83.68 (1.82)	79.47 (4.57)

^a^AUC: area under the receiver operating characteristic curve.

**Table 3:** Importance of each variable in the artificial neural network model.

Predictors	Values
Left ventricular ejection fraction	0.27
The number of implanted stents	0.14
Age	0.13
Diabetes	0.10
The number of vessels with coronary artery disease	0.09
Vascular disease	0.08
Brain natriuretic peptide	0.05
Glucose	0.05
Beta 2 microglobulin	0.04
Abnormal Q wave	0.02

## Discussion

### Principal Findings

In this study, we developed a machine learning–based model integrating clinical, anatomical, and laboratory test features to predict MACEs in patients with newly diagnosed AMI after their first PCI. The major findings suggest that the ANN model had higher predictive accuracy (accuracy of 87.99%, AUC of 0.81, and *F*_1_-score of 0.71), compared to RF, KNN, SVM, and logistic regression.

Among the patients with AMI in this study, the rates of MACEs at 30 days, 6 months, 1 year, and 3 years after PCI were 4.63%, 11.38%, 14.54%, and 18.50%, respectively, and the incidence of MACEs at 30 days after PCI was slightly less than the 5.5% reported in the Harmonizing Outcomes with RevascularIZatiON and Stents in Acute Myocardial Infarction study (HORIZONS-AMI) [25]. The incidence of MACEs at half a year was higher than the 6.67% reported by Chow et al [26], consistent with the 2-year rate of MACEs reported by Sanmenxia City (18.06%). The survival condition of patients with AMI after PCI was slightly different from that in other studies. The participants in this study were all patients who were first diagnosed with AMI and underwent PCI for the first time, and their prognosis was better than that of patients with previous myocardial infarctions and multiple PCIs [27]. In addition, the progression of a patient’s disease is affected by not only individual differences but also access to medical resources and services. The HORIZONS-AMI trial was first reported in 2012. Although the treatment level in the HORIZONS-AMI trial was higher than that available in China at that time, with the development of China’s economy, the progress of science and technology, and the substantial improvement of medical care, the MACE rate obtained in our study was lower than that reported in the HORIZONS-AMI study.

One study found that machine learning demonstrated the highest performance for risk prediction in patients with extracardiac vascular disease for the prediction of both arrhythmogenic cardiomyopathy and MACEs [10]. McCord et al [28] proposed that machine learning can be used to assess AMI within 30 minutes and that the algorithm has high diagnostic and prognostic utility. In this study, 3 algorithms were used to predict MACE occurrence for patients with newly diagnosed AMI undergoing PCI treatment for the first time. The MACE prediction ability of the logistic regression model was lower than that of the ANN model and almost the same as that of the RF model. However, the positive predictive values of these 3 prediction models were not high. Kuang et al [29] also found that the ANN model had the best predictive value for the transition from mild cognitive impairment to Alzheimer disease with ideal stability [29]. The positive predictive values of the RF model and the logistic regression model were both approximately 50%, which means that their predictive ability for MACEs was poor. Their shortcomings may be associated with class imbalances [30], which can easily cause the predicted results to be biased toward a large number of classes (the positive type of fault can be placed into the negative class). ANNs, with their powerful self-adaptability, self-organization, fault tolerance, and “black box” operation of nonlinear mapping, are especially suitable for solving problems with complex internal mechanisms and have been widely used in various disciplines [31].

Our results indicated that the 3-year prognostic risk among patients with AMI undergoing their first PCI was mainly related to age, ECG characteristics, ventricular ejection ability, coronary artery lesions, stent implantation after PCI, and some serological variables. Yang et al [32] found that the risk ratio of hospital deaths after PCI was 3.723 (95% CI 2.86-4.84) for South Korean patients aged >65 years relative to those aged ≤65 years. A Korean multicenter AMI National Institutes of Health–registered project found that the MACE rate, 3 years after PCI, among patients with AMI with an LVEF <40% was 3.34 times that of the control group [33]. Fam et al [34] conducted a retrospective study on patients with clinical AMI in Asian multiethnic groups and found that the risk of MACEs among patients with diabetes, 2 years after PCI, was 1.84 times higher than that among patients without diabetes [34]. Diabetes is a chronic metabolic disease, and long-term diabetes is often accompanied by bleeding disorders, vascular endothelial dysfunction, small artery lesions, high blood sugar [35], hemostatic disorders [36], endothelial dysfunction, and a series of other changes [37]. These characteristics will accelerate the process of atherosclerotic disease deterioration. The number of coronary artery lesions and the number of stents implanted in a patient are also positively correlated with the risk of postoperative MACEs to a certain extent. This may be because a higher number of vessels with coronary artery disease and the number of implanted stents tend to indicate a more serious condition, leading to a worse prognosis for the patients. Hongbo et al [38] found that the probability of a poor prognosis in patients with multiple coronary artery lesions was 20.0%, compared with 6.98% in patients with single coronary artery lesions [38].

The results of the machine learning model showed that predictors like LVEF, number of implanted stents, and age were more important to the model. LVEF is a common variable that reflects left ventricular function, and patients with a low LVEF have a significantly higher MACE rate [39]. An increase in age can lead to the aggravation of atherosclerosis [40]. The number of implanted stents may be related to the severity of the disease and the extent of the infarction [41]. This reminds us that we should pay special attention to the prognosis of patients with AMI who have a low LVEF value, older age, and more implanted stents in clinical practice.

### Study Limitations

This study has some limitations. First, there may have been an issue of survival bias in the study, as patients with missing follow-up data were excluded. Second, the data have missing values. We have filled missing values with multiple imputation; however, imputation with these techniques could synthetically reduce the variance in these variables and may have affected the accuracy of the constructed model. Finally, although the models were internally validated with data from the same hospital, further work should include validation with external data from other hospitals or centers.

### Conclusions

This study revealed that the ANN model showed good MACE prediction performance for patients with AMI after PCI, and it identified the most important predictors, which may aid in clinical decision-making and improve outcomes. This model needs to be externally validated in larger populations and multicenter settings.

## Appendix

Multimedia Appendix 1 Additional statistics.

## Abbreviations

AMI: acute myocardial infarction
ANN: artificial neural network
AUC: area under the receiver operating characteristic curve
ECG: electrocardiogram
HCM: hypertrophic cardiomyopathy
HORIZONS-AMI: Harmonizing Outcomes with RevascularIZatiON and Stents in Acute Myocardial Infarction
KNN: k-nearest neighbors
LVEF: left ventricular ejection fraction
MACE: major adverse cardiovascular event
PCI: percutaneous coronary intervention
RF: random forest
SVM: support vector machine

## Data Availability

The data sets generated and analyzed during this study are available from the corresponding author upon reasonable request.
