# Supplementary material for: Machine Learning for Early Prediction of Major Adverse Cardiovascular Events After First Percutaneous Coronary Intervention in Patients With Acute Myocardial Infarction: Retrospective Cohort Study
Source: JMIR Form Res. 2024 Jan 3;8:e48487. doi: 10.2196/48487 (PMC10794958; doi:10.2196/48487)
Supplement: Multimedia Appendix 1 [file formative_v8i1e48487_app1.docx]

**Table S1. Basic information of patients with AMI (n=1362)**

| **Characteristics** | | **N of**  **follow-up** | **Constitute**  **（%）** | **MACE** | | ***$\chi$^2^*** | ***P*** |
| --- | --- | --- | --- | --- | --- | --- | --- |
|  |  |  |  | **n** | ***%*** |  |  |
| gender | male | 1086 | 79.74 | 203 | 18.69 | 0.129 | .720 |
|  | female | 276 | 20.26 | 49 | 17.75 |  |  |
| Age | <65 | 644 | 47.28 | 101 | 15.70 | -2.070^▲^ | **.038** |
|  | 65- | 426 | 31.28 | 94 | 22.10 |  |  |
|  | 75- | 292 | 21.44 | 57 | 19.50 |  |  |
| BMI  classification | <18.5 | 42 | 3.08 | 10 | 23.81 | -0.902^▲^ | .367 |
|  | 18.5- | 649 | 47.65 | 109 | 16.80 |  |  |
|  | 24.0- | 573 | 42.07 | 115 | 20.07 |  |  |
|  | 28.0- | 98 | 7.20 | 18 | 18.37 |  |  |
| drinking | yes | 314 | 23.05 | 61 | 19.43 | 0.231 | .631 |
|  | no | 1048 | 76.95 | 191 | 18.23 |  |  |
| Smoking | yes | 509 | 37.38 | 93 | 18.27 | 3.221 | .200 |
|  | former | 54 | 3.96 | 15 | 27.78 |  |  |
|  | no | 799 | 58.66 | 144 | 18.02 |  |  |
| **Comorbidities** |  |  |  |  |  |  |  |
| Hypertension | yes | 663 | 48.68 | 125 | 18.85 | 0.106 | .745 |
|  | no | 699 | 51.32 | 127 | 18.17 |  |  |
| hyperlipidemia | yes | 388 | 28.49 | 70 | 18.04 | 0.076 | .782 |
|  | no | 974 | 71.51 | 182 | 18.69 |  |  |
| diabetes | yes | 336 | 24.67 | 75 | 22.32 | 4.315 | **.038** |
|  | no | 1026 | 75.33 | 177 | 17.25 |  |  |
| Cerebrovascular  disease | yes | 680 | 49.93 | 111 | 16.32 | 4.275 | **.039** |
|  | no | 682 | 50.07 | 141 | 20.67 |  |  |
| **Family** |  |  |  |  |  |  |  |
| diabetes | yes | 39 | 2.86 | 7 | 17.95 | 0.008 | .928 |
|  | no | 1323 | 97.14 | 245 | 18.52 |  |  |
| coronary heart  disease | yes | 21 | 1.54 | 4 | 19.05 | 0.004 | .948 |
|  | no | 1341 | 98.46 | 248 | 18.49 |  |  |
| Combined |  | 1362 | 100.00 | 252 | 18.50 |  |  |

▲ represents the results of Wilcoxon rank sum test.

**Table S2. Preoperative analysis of general conditions patients with AMI (n=1362)**

| **Characteristics** | | **n of**  **follow-up** | **Constitute**  **（%）** | **MACE** | | ***$\chi$^2^*** | ***P*** |
| --- | --- | --- | --- | --- | --- | --- | --- |
|  |  |  |  | n | % |  |  |
| Systolic | <90 | 60 | 4.41 | 11 | 18.33 | -0.065^▲^ | .948 |
| （mmHg） | 90- | 994 | 72.98 | 183 | 18.41 |  |  |
|  | 140- | 218 | 16.01 | 44 | 20.18 |  |  |
|  | 160- | 74 | 5.43 | 11 | 14.86 |  |  |
|  | 180- | 13 | 0.95 | 2 | 15.38 |  |  |
|  | 200- | 3 | 0.22 | 1 | 33.33 |  |  |
| Diastolic | <60 | 147 | 10.79 | 31 | 21.09 | -1.477^▲^ | .140 |
| （mmHg） | 60- | 998 | 73.27 | 188 | 18.84 |  |  |
|  | 90- | 145 | 10.65 | 22 | 15.17 |  |  |
|  | 100- | 51 | 3.74 | 7 | 13.73 |  |  |
|  | 110- | 18 | 1.32 | 2 | 11.11 |  |  |
|  | 120- | 3 | 0.22 | 2 | 66.67 |  |  |
| Heart rate | <60 | 89 | 6.53 | 14 | 15.73 | -0.354^▲^ | .723 |
| (times/min) | 60- | 1103 | 80.98 | 211 | 19.13 |  |  |
|  | 100- | 170 | 12.48 | 27 | 15.88 |  |  |
| Time from  onset to PCI（h） | <3 | 112 | 8.22 | 18 | 16.07 | -1.485^▲^ | .137 |
|  | 3- | 209 | 15.35 | 35 | 16.75 |  |  |
|  | 6- | 128 | 9.40 | 20 | 15.63 |  |  |
|  | 9- | 83 | 6.09 | 15 | 18.07 |  |  |
|  | 12- | 830 | 60.94 | 164 | 19.76 |  |  |
| Cardiac arrest | yes | 12 | 0.88 | 1 | 8.33 | 0.830 | .362 |
|  | no | 1350 | 99.12 | 251 | 18.59 |  |  |
| Pre-hospital thrombolysis | yes | 15 | 1.10 | 2 | 13.33 | 0.269 | .604 |
|  | no | 1347 | 98.90 | 250 | 18.56 |  |  |
| Pre-hospital defibrillation | yes | 5 | 0.37 | 1 | 20.00 | 0.007 | .931 |
|  | no | 1357 | 99.63 | 251 | 18.50 |  |  |
| AMI parting | STEMI | 455 | 33.41 | 87 | 19.12 | 0.173 | .677 |
|  | NSTEMI | 907 | 66.59 | 165 | 18.19 |  |  |
| Combined |  | 1362 | 100.00 | 252 | 18.50 |  |  |

▲ represents the results of Wilcoxon rank sum test.

**Table S3. Analysis of electrocardiogram, LVEF and Killip grading in patients with AMI (n=1362)**

| **Characteristics** | | **n of**  **follow-up** | **Constitute（%）** | **MACE** | | ***$\chi$^2^*** | ***P*** |
| --- | --- | --- | --- | --- | --- | --- | --- |
|  |  |  |  | n | % |  |  |
| Rhythm of the heart |  |  |  |  |  |  |  |
| Sinus rhythm | yes | 1278 | 93.83 | 238 | 18.62 | 0.200 | .655 |
|  | no | 84 | 6.17 | 14 | 16.67 |  |  |
| Atrial fibrillation | yes | 40 | 2.94 | 8 | 20.00 | 0.061 | .804 |
|  | no | 1322 | 97.06 | 244 | 18.46 |  |  |
| Pacemaker rhythm of the heart | yes | 25 | 1.84 | 6 | 24.00 | 0.510 | .475 |
|  | no | 1337 | 98.16 | 246 | 18.40 |  |  |
| Ⅲ degree or high atrioventricular block | yes | 22 | 1.62 | 2 | 9.09 | 1.313 | .252 |
|  | no | 1340 | 98.38 | 250 | 18.66 |  |  |
| Characteristics of Electrocardiograph | | |  |  |  |  |  |
| ST segment changes | yes | 521 | 38.25 | 106 | 20.35 | 1.901 | .168 |
|  | no | 841 | 61.75 | 146 | 17.36 |  |  |
| Complete left bundle branch block | yes | 25 | 1.84 | 4 | 16.00 | 0.106 | .745 |
|  | no | 1337 | 98.16 | 248 | 18.55 |  |  |
| Complete right bundle branch block | yes | 66 | 4.85 | 12 | 18.18 | 0.005 | .945 |
|  | no | 1296 | 95.15 | 240 | 18.52 |  |  |
| Abnormal Q wave | yes | 605 | 44.42 | 125 | 20.66 | 4.254 | **.039** |
|  | no | 757 | 55.58 | 127 | 16.78 |  |  |
| **LVEF** | >50% | 999 | 73.35 | 167 | 16.72 | -2.812^▲^ | **.005** |
|  | 40%~50% | 245 | 17.99 | 57 | 23.27 |  |  |
|  | 30%~40% | 84 | 6.17 | 19 | 22.62 |  |  |
|  | <30% | 34 | 2.50 | 9 | 26.47 |  |  |
| **Kiliip** | Ⅰ | 177 | 13.00 | 31 | 17.51 | -0.436^▲^ | .663 |
|  | Ⅱ | 820 | 60.21 | 160 | 19.51 |  |  |
|  | Ⅲ | 297 | 21.81 | 45 | 15.15 |  |  |
|  | Ⅳ | 68 | 4.99 | 16 | 23.53 |  |  |
| Combined |  | 1362 | 100.00 | 252 | 18.50 |  |  |

▲ represents the results of Wilcoxon rank sum test.

LVEF, left ventricular ejection fraction；

Table S4. Analysis of infarct site in patients with AMI (n=1362)

| **Characteristics** | | **N of**  **follow-up** | **Constitute**  **（%）** | **MACE** | | ***$\chi$^2^*** | ***P*** |
| --- | --- | --- | --- | --- | --- | --- | --- |
|  |  |  |  | **n** | ***%*** |  |  |
| Before the partition | yes | 115 | 8.44 | 22 | 19.13 | 0.033 | .856 |
|  | no | 1247 | 91.56 | 230 | 18.44 |  |  |
| anterior wall | yes | 475 | 34.88 | 93 | 19.58 | 0.561 | .454 |
|  | no | 887 | 65.12 | 159 | 17.93 |  |  |
| Extensive anterior | yes | 133 | 9.77 | 28 | 21.05 | 0.636 | .425 |
|  | no | 1229 | 90.23 | 224 | 18.23 |  |  |
| high lateral wall | yes | 56 | 4.11 | 15 | 26.79 | 2.658 | .103 |
|  | no | 1306 | 95.89 | 237 | 18.15 |  |  |
| anterior lateral wall | yes | 19 | 1.40 | 2 | 10.53 | 0.813 | .367 |
|  | no | 1343 | 98.60 | 250 | 18.62 |  |  |
| lower wall | yes | 585 | 42.95 | 107 | 18.29 | 0.030 | .861 |
|  | no | 777 | 57.05 | 145 | 18.66 |  |  |
| positive posterior wall | yes | 55 | 4.04 | 6 | 10.91 | 2.191 | .139 |
|  | no | 1307 | 95.96 | 246 | 18.82 |  |  |
| Combined |  | 1362 | 100.00 | 252 | 18.57 |  |  |

Anterior wall (V1-V2 lead), anterior wall (V1-V3, V4 lead), extensive anterior wall (V1-V5, V6 lead), high lateral wall (Ⅰ, AVL lead), anterior lateral wall (V5, V6 lead), lower wall (on, on, AVF lead), positive posterior wall (V7-V9 lead).

**Table S5. PCI operation in patients with AMI (n=1362)**

| **Characteristics** | | **N of follow** | **Constitute（*%*）** | **MACE** | | *$\chi$****^2^*** | ***P*** |
| --- | --- | --- | --- | --- | --- | --- | --- |
|  |  |  |  | **n** | **%** |  |  |
| **Coronary angiography** |  |  |  |  |  |  |  |
| Number of vessels with coronary artery disease | Ⅰ | 333 | 24.45 | 45 | 13.51 | -3.497^▲^ | **<.001** |
|  | Ⅱ | 445 | 32.67 | 75 | 16.85 |  |  |
|  | Ⅲ | 541 | 39.72 | 123 | 22.74 |  |  |
|  | Ⅳ | 43 | 3.16 | 9 | 20.93 |  |  |
| Classification of pathological stenosis | mild | 4 | 0.29 | 0 | 0.00 | -1.649^▲^ | .099 |
|  | moderate | 11 | 0.81 | 1 | 9.09 |  |  |
|  | severe | 278 | 20.41 | 44 | 15.83 |  |  |
|  | very severe severely | 355 | 26.06 | 65 | 18.31 |  |  |
|  | occlusion | 714 | 52.42 | 142 | 19.89 |  |  |
| **Operation method** |  |  |  |  |  |  |  |
| Thrombus suction | yes | 206 | 15.12 | 36 | 17.48 | 0.170 | .680 |
|  | no | 1156 | 84.88 | 216 | 18.69 |  |  |
| Stent implantation | yes | 1312 | 96.33 | 242 | 18.45 | 0.616 | .433 |
|  | no | 50 | 3.67 | 10 | 20.00 |  |  |
| Implanted stent number | No | 50 | 3.67 | 10 | 20.00 | -2.876^▲^ | **.004** |
|  | Ⅰ | 700 | 51.40 | 106 | 15.14 |  |  |
|  | Ⅱ | 385 | 28.27 | 84 | 21.82 |  |  |
|  | Ⅲ | 151 | 11.09 | 37 | 24.50 |  |  |
|  | ≥Ⅳ | 76 | 5.58 | 15 | 19.74 |  |  |
| **Combined** |  | 1357 | 100.00 | 252 | 18.57 |  |  |

▲ represents the results of Wilcoxon rank sum test.

**Table S6. Analysis of blood test indexes in patients with AMI (n=1362)**

| **Measurements** | **MACE（*n*=252）** | | |  | **NO-MACE (*n*=1110)** | | | ***t*** | ***P*** |
| --- | --- | --- | --- | --- | --- | --- | --- | --- | --- |
|  | **Min** | **Max** | **Mean(SD)** |  | **Min** | **Max** | **Mean(SD)** |  |  |
| **Blood routine test** | |  |  |  |  |  |  |  |  |
| WBC(10^9^/L) | 3.53 | 21.35 | 9.02(3.36) |  | 1.67 | 24.99 | 9.17(3.36) | 0.630 | .529 |
| Hb(g/L) | 34.00 | 175.00 | 128.19(20.34) |  | 52.00 | 180.00 | 128.39(21.07) | 0.136 | .892 |
| HCT(%) | 18.80 | 52.60 | 39.21(5.68) |  | 12.70 | 54.80 | 38.92(6.13) | -0.680 | .497 |
| hs-cTn(ng/mL) | 0.01 | 53.84 | 20.26(19.59) |  | 0.01 | 77.22 | 21.44(19.68) | 0.856 | .392 |
| BNP(pg/μL) | 2.37 | 6677.80 | 684.36(997.9) |  | 2.00 | 6095.90 | 518.27(773.65) | -2.478 | **.014** |
| HBA1C(%) | 0.50 | 13.00 | 6.44(1.48) |  | 1.50 | 15.40 | 6.32(1.30) | -1.151 | .251 |
| K^+^(mmol/L) | 2.89 | 6.77 | 3.92(0.54) |  | 2.50 | 7.77 | 3.9(0.53) | -0.618 | .526 |
| **Myocardial enzyme spectrogram + liver function test** | | | | | | | | | |
| aHBDH(U/L) | 1.50 | 1920.90 | 301.91(297.62) |  | 1.50 | 2687.17 | 306.46(284) | 0.227 | .820 |
| AST(U/L) | 12.50 | 4833.00 | 133.87(337.13) |  | 4.90 | 7750.50 | 128.49(284.31) | -0.223 | .824 |
| CK(U/L) | 25.59 | 17997.60 | 962.82(1870.28) |  | 22.66 | 23945.41 | 839.94(1489.82) | -0.975 | .330 |
| CK-MB(U/L) | 2.53 | 4395.02 | 115.9(341.45) |  | 0.21 | 11516.00 | 99.51(465.99) | -0.639 | .598 |
| LDH(U/L) | 49.28 | 6455.62 | 442.78(505.02) |  | 25.97 | 6686.24 | 431(447.85) | -0.368 | .713 |
| Mb(μg/L) | 20.98 | 9350.26 | 396.72(860.38) |  | 1.53 | 9596.53 | 439.19(838.7) | 0.722 | .470 |
| **Renal function** |  |  |  |  |  |  |  |  |  |
| Cre(mmol/L) | 44.45 | 1318.19 | 113.62(106.82) |  | 7.55 | 1470.05 | 103.68(88.44) | -1.548 | .122 |
| Scr(mL/min) | 5.04 | 170.70 | 65.19(30.18) |  | 3.21 | 722.03 | 71.87(44.35) | 2.274 | **.023** |
| eGFR(ml/min) | 3.61 | 171.74 | 75.68(28.92) |  | 1.58 | 252.45 | 80.55(31.82) | 2.229 | **.026** |
| RBP(mg/L) | 1.98 | 274.30 | 38.28(22.29) |  | 0.10 | 255.65 | 37.81(18.09) | -0.832 | .406 |
| β2-MG(mg/L) | 0.59 | 33.85 | 3.23(3.61) |  | 0.25 | 35.75 | 2.72(5.51) | -2.670 | **.034** |
| **Blood fat check** |  |  |  |  |  |  |  |  |  |
| CHOL(mmol/L) | 0.55 | 10.21 | 4.53(1.23) |  | 0.24 | 11.76 | 4.58(1.33) | 0.536 | .592 |
| TG(mmol/L) | 0.36 | 9.36 | 1.59(1.08) |  | 0.14 | 22.61 | 1.7(1.50) | 1.198 | .232 |
| HDL-c(mmol/L) | 0.30 | 1.81 | 1.04(0.28) |  | 0.05 | 8.27 | 1.06(0.38) | 1.111 | .267 |
| LDL-c(mmol/L) | 0.90 | 7.03 | 2.9(1.02) |  | 0.57 | 8.31 | 2.94(1.12) | 0.489 | .625 |
| GLU(mmol/L) | 3.60 | 21.61 | 7.22(3.32) |  | 2.87 | 48.29 | 6.68(3.00) | -2.381 | **.018** |

WBC, white blood cell; Hb, hemoglobin; HCT, hematocrit; Hs-ctn, hypersensitive troponin; BNP, B-type brain natriuretic peptide; HbA1c, glycosylated hemoglobin; K+, potassium; AHBDH, α hydroxy butyric acid; AST, aspartic acid; CK, creatine kinase; CK-MB, creatine kinase isoenzyme; LDH, lactate dehydrogenase; MB, myoglobin; CRE, serum creatinine; SCR, serum creatinine clearance; EGFR, estimated glomerular filtration rate; RBP, retinol binding protein; β2-Mg, β2 microglobulin; Chol, total cholesterol; TG, triglyceride; HDL-C, high-density lipoprotein; LDL-C, low density lipoprotein; Glu, glucose
